# Individual Differences in Laughter Perception Reveal Roles for Mentalizing and Sensorimotor Systems in the Evaluation of Emotional Authenticity

**DOI:** 10.1093/cercor/bht227

**Published:** 2013-08-22

**Authors:** C. McGettigan, E. Walsh, R. Jessop, Z. K. Agnew, D. A. Sauter, J. E. Warren, S. K. Scott

**Affiliations:** 1 Department of Psychology, Royal Holloway University of London, Egham TW20 0EX, UK,; 2 Institute of Cognitive Neuroscience, University College London, London WC1N 3AR, UK,; 3 Institute of Psychiatry, King's College London, London SE5 8AF, UK,; 4 Department of Social Psychology, University of Amsterdam, 1018 XA Amsterdam, Netherlands and; 5 Department of Cognitive Perceptual and Brain Sciences, University College London, London WC1H 0AP, UK

**Keywords:** emotion, functional MRI, laughter, medial prefrontal cortex, sensorimotor cortex

## Abstract

Humans express laughter differently depending on the context: polite titters of agreement are very different from explosions of mirth. Using functional MRI, we explored the neural responses during passive listening to authentic amusement laughter and controlled, voluntary laughter. We found greater activity in anterior medial prefrontal cortex (amPFC) to the deliberate, Emitted Laughs, suggesting an obligatory attempt to determine others' mental states when laughter is perceived as less genuine. In contrast, passive perception of authentic Evoked Laughs was associated with greater activity in bilateral superior temporal gyri. An individual differences analysis found that greater accuracy on a post hoc test of authenticity judgments of laughter predicted the magnitude of passive listening responses to laughter in amPFC, as well as several regions in sensorimotor cortex (in line with simulation accounts of emotion perception). These medial prefrontal and sensorimotor sites showed enhanced positive connectivity with cortical and subcortical regions during listening to involuntary laughter, indicating a complex set of interacting systems supporting the automatic emotional evaluation of heard vocalizations.

## Introduction

Historically, psychology and cognitive neuroscience have focused on the perception of negative emotions (Fredrickson 1998). However, in recent years, there has been increasing interest in characterizing the perception of positive emotions, including laughter. Laughter has been identified in several mammal species (Panksepp 2000, 2005; Panksepp and Burgdorf 2000, 2003; Ross et al. 2009, 2010; Davila-Ross et al. 2011), and in humans was found to be the only positive vocal emotional expression recognized across culturally and geographically distinct groups (Sauter et al. 2010). The spontaneous laughter seen when chimpanzees are tickled or playing differs from that in response to the laughter of other chimpanzees (Davila-Ross et al. 2011). This acoustic difference reflects a functional difference: the laughter elicited by others' laughter is associated with attempts to sustain and prolong social play, and play lasts longer when laughter is echoed. Davila-Ross and coworkers compared this pattern to variable expressions of laughter in human interactions, where laughter is predominantly used as a social glue to promote and maintain affiliations and group membership.

### More than One Way to Laugh

Several authors have described and characterized various types of laughter in humans (Wild et al. 2003; Gervais and Wilson 2005; Szameitat, Alter, Szameitat, Darwin et al. 2009, Szameitat, Alter, Szameitat, Wildgruber et al. 2009, 2010, 2011; Wattendorf et al. 2012). Szameitat and coworkers have shown that different laughter categories have varying acoustic properties (e.g., laughter during tickling, versus taunting and schadenfreude laughter; Szameitat, Alter, Szameitat, Wildgruber et al. 2009), can be accurately classified by listeners, and are perceived to have different emotional qualities (Szameitat, Alter, Szameitat, Darwin et al. 2009). Further, it has been shown using functional MRI (fMRI) that neural responses during laughter perception differ depending on the category of laughter heard (Szameitat et al. 2010). These classifications of types of laughter (with associated variation in emotional meaning) make the prediction that any one laugh will have a particular meaning (e.g., a joyful laugh will signal joy), without accounting for the ways that laughter, as a social cue, can have different senses (positive or negative) depending on context (Scott 2013). Furthermore, all of these previous studies investigated laughter perception using stimuli produced by actors, which were all to some extent posed, meaning that none of these studies were designed to address uncontrolled, authentic laughter (nor how this is perceived). In detailed review articles, both Wild et al. (2003) and Gervais and Wilson (2005) draw upon a wealth of behavioral, neuropsychological, and neurological data to distinguish between “voluntary” and “involuntary” laughter in humans. Gervais and Wilson (2005) describe involuntary, uncontrolled laughter as “stimulus driven and emotionally valenced” (p. 403), and associated with mirthful vocalizations. In contrast, they claim that voluntary laughter may not necessarily be associated with a particular emotional experience, and could rather perform a variety of social functions like signaling affiliation or polite agreement in conversation (Smoski and Bachorowski 2003; Gervais and Wilson 2005). Indeed, an acoustic analysis of conversations by Vettin and Todt (2004) indicated that social laughter (analogous to Gervais and Wilson's “voluntary” laughter) occurs very frequently in this context, and possesses different acoustic characteristics from stimulus-driven laughter. In terms of the production of laughter, a recent functional imaging study by Wattendorf et al. (2012) identified differences in the profile of neural activation seen during the involuntary laughter evoked by tickling, where these laughs were associated with greater signal in the hypothalamus compared with voluntary laughter that was emitted “on demand” by the participants.

Characterizing the effects of variable voluntary control on the perception of laughter, and the neural correlates of this, is crucial to developing a greater understanding of laughter as a vocalization used often and flexibly in human communication (Provine 2000). More generally, the distinction between voluntary and involuntary control of emotional vocalizations in the laboratory can also address a comparison of acted/posed and authentic expressions of felt emotion. This is relevant for the wider field of emotion research, in which, for ethical and practical reasons (consider emotions such as fear, disgust, anger), the expressions used as stimuli are typically posed or acted.

### Understanding Laughter in the Brain–Contagion and the Role of Sensorimotor Systems

In a previous fMRI study, we identified that activity in regions of sensorimotor cortex involved in orofacial smiling movements correlated positively with valence and arousal during passive listening to nonverbal vocalizations (including sounds of fear, disgust, amusement, and triumph; (Warren et al. 2006). As the more positive vocalizations (laughter and cheering) are typically expressed in groups—laughter is 30 times more likely to occur in the presence of others than in a solo setting (Provine 2000)—we attributed specific activations in lateral sensorimotor cortex to a facilitation for vocalizations promoting social cohesion in primate groups (Warren et al. 2006). The current study aims to refine our understanding of the role of sensorimotor cortex in the perception of positive emotions. Specifically, we hypothesized that if cortical motor and somatosensory facilitation is an index of contagion, then activation in response to heard laughter should be modulated by its contagiousness, that is, more infectious laughter should elicit a greater motor readiness to join in. However, if, as suggested by simulation accounts, the role of sensorimotor cortex in the perception of social cues is to support a higher-order mechanism for the social and emotional understanding of others (Carr et al. 2003), there might be no such straightforward relationship between laughter contagion and facilitation.

### The Current Study

We designed an fMRI study to address 2 novel questions related to the perception of emotional vocalizations. First, we aimed to conduct the first direct investigation of the neural correlates of perceived emotional authenticity in heard nonverbal vocalizations. Similar to a recent study of the production of ticklish laughter (Wattendorf et al. 2012), we took advantage of the fact that laughter can be evoked from humans harmlessly and relatively easily, but can also be readily acted or posed. We elicited tokens of genuine amusement laughter (henceforth Evoked laughter) by showing humorous audiovisual stimuli to speakers of British English. Using the same talkers, we also recorded deliberate, voluntary laughs (henceforth Emitted laughter) in the absence of humorous stimuli. In behavioral pilot testing, we found that naïve listeners performed significantly better than chance in classifying the recorded laughs as “real” (Evoked) or “posed” (Emitted), in line with how these laughs were produced—as an expression of genuine amusement, or not. The Evoked laughs were perceived to be more contagious—both behaviorally and emotionally—than the Emitted laughter. This finding allowed us to address our second aim—to test the prediction that more genuine expressions of positive emotion are behaviorally more contagious, and therefore should yield stronger engagement of sensorimotor cortex, in support of a facilitation account of group vocalization behavior.

In a recent review, Brueck et al. (2011) caution that affective processing is particularly subject to idiosyncrasies in the perceiver, which may be transient and mood dependent, or rather more stable in the individual (e.g., age or personality-related). They suggest that individual variability in emotion perception is underexploited in the literature, and may yield insights that have so far been masked by traditional group-averaging approaches. We acknowledge that the perception of authenticity in laughter is potentially a highly subjective process that may vary considerably across listeners—thus, in addressing the above aims, we endeavored to adopt an approach more driven by individual differences, taking the investigation of neural correlates of laughter perception beyond the group-averaging approaches favored in previous work (Warren et al. 2006; Szameitat et al. 2010).

## Materials and Methods

### Stimuli

The emotional vocalization stimuli were generated by 3 female speakers of British English (aged 28, 29, and 43 years). Stimuli were recorded in a sound-proof, anechoic chamber. Recordings were made on a digital audio tape recorder (Sony 60ES; Sony UK Limited, Weybridge, UK) and fed to the S/PDIF digital input of a PC soundcard (M-Audio Delta 66; M-Audio, Iver Heath, UK).

Three types of emotional vocalization were recorded in the order: Emitted Laughter, Evoked Laughter, Disgust. For Emitted Laughter, the speaker was instructed to simulate tokens of amusement laughter, in the absence of any external stimulation and without entering a genuine state of amusement. They were encouraged to make the laughter sound natural and positive. In order to avoid any carry-over of genuine amusement into the Emitted Laughter recordings, the recording of Emitted Laughter always preceded the Evoked Laughter phase. During the second part of the recording session, each speaker was allowed to watch video clips that she reported as finding highly amusing and that would typically to cause her to laugh aloud. These were presented from YouTube (www.youtube.com) on a computer monitor inside the chamber, with the audio track played over headphones. The speaker was encouraged to produce laughter freely and spontaneously in response to the video stimuli.

The Disgust sounds, which were posed, were included in the experiment as an emotional distractor condition, in order that the participants in the imaging study would be less likely to detect that the main experimental manipulation concerned the laughter only. The speakers attended a separate recording session and generated posed, nonverbal tokens of disgust, where they were asked to simulate the kind of sound one might make having seen or smelled something disgusting. As for the Emitted Laughter recording, these tokens were generated in the absence of external stimuli.

The audio files were downsampled at a rate of 44 100 Hz to mono .wav files with 16-bit resolution. These were further edited into separate .wav files containing short (<7 s each), natural epochs of laughter/disgust, using Audacity (http://audacity.sourceforge.net/). This process resulted in 65 tokens of Evoked laughter (Speaker A: 14 tokens, Speaker B: 32, Speaker C: 19 tokens; mean duration: 4.14 s), 60 tokens of Emitted laughter (Speaker A: 17 tokens, Speaker B: 17 tokens, Speaker C: 26 tokens; mean duration 2.98 s), and 52 tokens of Disgust (Speaker A: 16 tokens, Speaker B: 16 tokens, Speaker C: 19 tokens; mean duration 1.70 s).

In order to select the best examples from the Evoked and Emitted laughter tokens, these were presented to 4 independent raters who categorized each token as “Real” or “Posed.” The items were presented individually, in a random order, using MATLAB (The Mathworks, Natick, MA, USA) with the Cogent toolbox extension (www.vislab.ucl.ac.uk/Cogent/). The raters listened to the stimuli over Sennheiser HD201 headphones (Sennheiser UK, High Wycombe, Buckinghamshire, UK). Responses were made by a key press after each stimulus and progress through the experiment was self-timed. Only those stimuli that were labeled accurately by at least 3 of 4 raters were selected for use in behavioral testing. This selection process resulted in 21 examples of Evoked laughs (Speaker A: 6 tokens, Speaker B: 8 tokens, Speaker C: 7 tokens) and 21 Emitted laughs (Speaker A: 8 tokens, Speaker B: 6 tokens, Speaker C: 7 tokens) for use in the final experiment. The Evoked laughs had a mean duration of 3.24 s (SD 1.54), and the Emitted laughs had a mean duration of 2.62 s (SD 1.05).

### Pilot Testing I: Classification of Evoked and Emitted Laughter Tokens

Seventeen adult participants (9 females) completed a classification test on the 21 Evoked and 21 Emitted laughter tokens in the same procedure used in the initial selection process above. The group classified the stimuli with 80.4% accuracy (mean *d*′: 2.01). There was no significant difference in the hit rate for Evoked (87%) and Emitted (75%) items (*t*_(16)_ = 1.875; *P* = 0.079), nor was there any difference in accuracy between female and male participants.

Before inclusion in the imaging experiment, the Evoked laughter tokens underwent further editing to truncate silent periods, in order that the 2 laughter categories were no longer significantly different in duration (New mean duration of Evoked laughs: 3.06 s). Twenty-one separate Disgust tokens (Speaker A: 8, Speaker B: 6, Speaker C: 7; mean duration 2.64 s) were selected by the experimenters and added to the stimulus set. A fourth condition, intended as a low-emotion distractor set, was constructed by manually combining parts of all 3 emotion conditions, within-speaker, to create 21 “mixed” stimuli (Speaker A: 8, Speaker B: 6, Speaker C: 7; mean duration 2.96 s). These combined items were low-pass filtered at 4 kHz and spectrally rotated around 2 kHz (in MATLAB; Blesser 1972) to render them unintelligible. The emotional conditions were also low-pass filtered at 4 kHz for consistency across conditions. Finally, all 84 tokens (21 from each condition) were normalized for peak amplitude in PRAAT (Boersma and Weenink 2010).

### Pilot Testing II: Emotional Ratings

Twenty adult participants (10 females) rated the 21 Evoked and 21 Emitted laughs, as well as the Disgust and unintelligible items, on 7-point Likert scales of Arousal, Intensity, Valence, and Contagiousness. There were 2 Contagion ratings: one for how much the sound made the listener feel they wanted to move their face (Behavioral Contagion) and the other describing how much the sound made the listener feel an emotion (Emotional Contagion). For the Arousal, Intensity, and Contagion ratings, the scale ranged from 1 (“Not at all arousing/intense/contagious”) to 7 (“Extremely arousing/intense/contagious”), where 4 represented moderate arousal/intensity/contagion. Here, the Intensity scale referred to the perceived emotional intensity of the vocalization (rather than its acoustic intensity). The Valence scale ranged from 1 being “Highly Negative” to 7 being “Highly Positive,” with 4 being “Neutral.”

The stimuli were presented using MATLAB (version R2010a), with the Cogent toolbox extension (www.vislab.ucl.ac.uk). The participants rated the laughter stimuli in blocks (one block for each rating scale), with block order, and within-block stimulus order randomized. In each experimental block, participants were presented with all 84 stimuli. At the end of each trial, the rating scale was displayed on the computer screen. The participant rated the laughter stimuli by key press.

On all 5 scales, the Evoked laughs received higher ratings than the Emitted laughs. This difference was significant for Intensity (Means: 4.13 and 3.58, *t*_(40)_ = 4.84, *P* < 0.0001), Valence (Means: 5.38 and 4.74, *t*_(40)_ = 6.19, *P* < 0.0001), Behavioral Contagion (Means: 3.91 and 3.43, *t*_(40)_ = 3.32, *P* < 0.005) and Emotional Contagion (Means: 4.13 and 3.58, *t*_(40)_ = 6.34, *P* < 0.0001), and marginally significant for Arousal (Means: 3.60 and 3.39, *t*_(32)_ = 2.00, *P* = 0.055; df modified for nonequal variance). Notably, both laughter types were rated as positively valenced (i.e., significantly >4 (neutral); Evoked: *t*_(20)_ = 25.82, *P* < 0.0001; Emitted: *t*_(20)_ = 17.23, *P* < 0.0001).

### Acoustic Properties of Evoked and Emitted Laughs

Using the phonetic analysis software PRAAT (Boersma and Weenink 2010), we extracted a range of basic acoustic parameters—duration (s), intensity (dB; not to be confused with the emotional Intensity scale used in Pilot II, described above), mean, minimum, maximum, and standard deviation of F0 (Hz), spectral center of gravity (Hz), and spectral standard deviation (Hz)—for each of the Evoked and Emitted laughs. Independent *t*-test comparisons showed that the 2 categories were significantly different in pitch (Mean F0: Evoked = 491.5 Hz (SD 113.8 Hz), Emitted = 326.1 Hz (SD 62.0 Hz), *t*_(40)_ = 5.85 *P* < 0.0001; Min F0: Evoked = 284.0 Hz (SD 136.8 Hz), Emitted = 167.0 Hz (SD 44.6 Hz), *t*_(40)_ = 3.73, *P* < 0.005; Max F0: Evoked = 752.5 Hz (SD 183.2 Hz), Emitted = 560.3 Hz (SD 194.0 Hz), *t*_(40)_ = 3.30, *P* < 0.005), but not on the other measures.

### Functional Magnetic Resonance Imaging

#### Participants

Twenty-one adult speakers of English (13 females; mean age 23 years 11 months) participated in the experiment. None of the participants had taken part in the pilot tests. All had healthy hearing and no history of neurological incidents, nor any problems with speech or language (self-reported). The study was approved by the UCL Research Ethics Committee.

#### Passive Listening to Laughter

Functional imaging data were acquired on a Siemens Avanto 1.5-Tesla MRI scanner (Siemens AG, Erlangen, Germany). Before going into the scanner, the participants were informed that they would hear emotional sounds and some other types of sound, and that they should listen carefully to these with their eyes closed. They were reminded that they should keep their head and face very still throughout the experiment. Aside from these instructions, the listeners were not required to perform any overt task and were not informed that the study was about laughter perception.

To check for changes in facial expression during the experiment, which may reflect contagiousness of the emotional stimuli, an in-bore camera was trained on the participant's face throughout. An experimenter watched the camera feed throughout the session and noted any movements of the mouth, nose, or eyes, by trial number. None of the participants was observed to smile or produce any recognizable non-neutral expression. Overall, there were so few movements observed during the passive listening phase, either within or across listeners, that no statistical analysis could be usefully performed on the data. Thus, the auditory stimuli did not lead to the production of overt orofacial responses in the listeners during the experiment.

Auditory presentation of emotional sounds took place in 2 runs of 110 echo-planar whole-brain volumes (TR = 9 s, TA = 3 s, TE = 50 ms, flip angle = 90°, 35 axial slices, 3 mm × 3 mm × 3 mm in-plane resolution). A sparse-sampling routine (Edmister et al. 1999; Hall et al. 1999) was employed, in which the auditory stimuli were presented in the quiet period between scans. Auditory onsets occurred 4.3 s (±0.5 s jitter) before the beginning of the next whole-brain volume acquisition. Auditory stimuli were presented using MATLAB with the Psychophysics Toolbox extension (Brainard 1997), via a Sony STR-DH510 digital AV control center (Sony, Basingstoke, UK) and MR-compatible insert earphones (Etymotic Research, Inc., Elk Grove Village, IL) worn by the participant.

All 84 stimuli (21 from each condition) were presented twice in total (once in each functional run). The condition order was pseudorandomized, with each auditory condition occurring once every 4 trials, separated by 5 evenly spaced mini-blocks of a Rest Baseline condition (each lasting 7 TRs).

#### Orofacial Movements Localizer

After the auditory phase of the experiment, the listeners were informed that the next part of the experiment would involve making movements of the face. Using PhotoBooth (Apple, Cupertino, CA, USA), live video footage of the experimenter in the control room was shown to the participant via a specially configured video projector (Eiki International, Inc., Rancho Santa Margarita, CA, USA). The images were projected onto a custom-built front screen, which the participant viewed via a mirror placed on the head coil. Using the audio intercom system, the experimenter was able to describe the upcoming task, and demonstrate the required facial movements.

The participant was told that they would be asked to make 2 different types of movement in the scanner, called “Smile” and “Wrinkle.” In the Smile condition, the participant was asked to alternate between a smiling and a neutral facial expression, at an alternation rate of about 1 s. In the Wrinkle condition, the participant was asked to wrinkle their nose (similar to an expression of disgust), in alternation with rest. A total of 125 echo-planar whole-brain volumes (TR = 3 s, TA = 3 s, TE = 50 msec, flip angle = 90°, 35 axial slices, 3 mm × 3 mm × 3 mm in-plane resolution) were acquired during the task, in which the participants performed 4 blocks of Smile, Wrinkle, and Rest (no movement). The blocks lasted 21 s each and were presented in a pseudorandom order, where each sequence of 3 blocks contained one block from each of the conditions. Each block was separated by 3 volumes, in which onscreen text instructed the participant to stop the current movement (STOP), prepare for the next trial (Get ready to SMILE/WRINKLE/REST), and start moving (GO), respectively. As in the auditory session, the experimenters watched the in-scanner camera feed to check that the participants were performing the task adequately.

After the localizer was complete, a high-resolution *T*_1_-weighted anatomical image was acquired (HIRes MP-RAGE, 160 sagittal slices, voxel size = 1 mm^3^). The total time in the scanner was around 50 min.

#### Behavioral Post-Test

After the scanning session was complete, the participants were informed that some of the laughs they heard in the scanner were genuine expressions of amusement, while others were posed. The participant was then asked to listen to each of the stimuli again and classify the items as “real” or “posed.” The stimuli were presented in a quiet room, using the same equipment and procedure as in the pilot classification experiment. Individual performances were calculated as *d*′ scores for use in analyses of the functional data.

#### Analysis of fMRI Data

Data were preprocessed and analyzed in SPM8 (Wellcome Trust Centre for Neuroimaging, London, UK). Functional images were realigned and unwarped, co-registered with the anatomical image, normalized using parameters obtained from unified segmentation of the anatomical image, and smoothed using a Gaussian kernel of 8 mm FWHM.

#### Auditory Session

At the single-subject level, event onsets from all 5 conditions (Evoked Laughter, Emitted Laughter, Disgust, Unintelligible Baseline, Rest Baseline) were modeled as instantaneous and convolved with the canonical hemodynamic response function. Contrast images were calculated to describe the comparisons Evoked Laughter > Emitted Laughter and All Laughs (Evoked and Emitted) > Rest Baseline. The Evoked Laughter > Emitted Laughter images were entered into a second-level, 1-sample *t*-tests for the group analysis. Additional second-level regression models were also run for each of the contrasts Evoked Laughter > Emitted Laughter, Emitted Laughter > Evoked Laughter and All Laughs > Rest, with individual *d*′ scores from the behavioral post-test as a covariate in each case.

To allow a comparison of perceived authenticity in laughter, the Evoked and Emitted conditions were recoded at the single-subject level according to each participant's post-test labels of “real” and “posed,” respectively. The first-level data were then analyzed as above, with group 1-sample *t*-tests to explore comparisons of “Real” > “Posed” and “Posed” > “Real.” A further second-level paired *t*-test was run to directly compare the “Real” > “Posed” with the Evoked > Emitted activations, and to compare the “Posed” > “Real” with the Emitted > Evoked contrast.

Using the MarsBaR toolbox (Brett et al. 2002), spherical regions of interest (ROIs) of 4 mm radius were built around the peak voxels in selected contrasts—parameter estimates were extracted from these ROIs and used to construct activation plots.

#### Orofacial Movements Localizer

For each subject, the 3 conditions Smile, Wrinkle, and Rest were modeled as events of duration 21 s and convolved with the canonical hemodynamic response function. Second-level contrast images for Smile > Rest were used to illustrate the overlap between perceptual responses to laughter (as found in the individual differences regression analyses) and brain regions supporting orofacial movements.

#### Functional Connectivity—Psychophysiological Interactions

Psychophysiological interaction (PPI) analyses were used to investigate changes in connectivity between selected seed regions and the rest of the brain that were dependent on the perceived authenticity of laughter. In each subject, the first eigenvariate of the BOLD time course was extracted from 4 seed volumes of interest (VOIs)—these were significant clusters in anterior medial prefrontal cortex (amPFC), left and right somatosensory cortex, and left presupplementary motor area (pre-SMA) from the second-level regression analysis of behavioral post-test scores against All Laughs > Rest. The sensorimotor clusters were selected based on our a priori hypothesis about a role for motor and somatosensory cortex in laughter perception, in order to interrogate the sensitivity of these regions to the 2 laughter categories: the 3 selected clusters were those that overlapped with regions activated by the orofacial movements localizer (Smile > Rest, plotted at a voxelwise height threshold of *P* < 0.001 (uncorrected)). For each VOI, a PPI regressor was built which described the interaction between the activation time course and a psychological regressor for the contrast of interest (in this case, the recoded conditions “Real” > “Posed”). The PPI was evaluated at the first level in a model with the individual physiological and psychological time courses included as covariates of no interest, followed by a random effects 1-sample *t*-test to investigate positive interactions based on the contrasts “Real” > “Posed” and “Posed” > “Real.”

All results of the subtraction contrasts in the experiment are reported at an uncorrected voxel height threshold of *P* < 0.001. The results of the regression and connectivity (PPI) analyses are reported at a voxel height threshold of *P* < 0.005 (uncorrected) in the interest of exploring the wider networks involved in individual differences and functional interactions. Except for the orofacial movements localizer contrast (which had no cluster threshold), a cluster extent correction was applied for a whole-brain alpha of *P* < 0.001, using a Monte Carlo simulation with 10 000 iterations implemented in MATLAB (Slotnick et al. 2003). This determined that an extent threshold of 20 voxels (where the probability curve approached 0) could be applied for both voxel height thresholds of *P* < 0.001 and *P* < 0.005. The anatomical locations of significant clusters (at least 8 mm apart) were labeled using the SPM Anatomy Toolbox (version 18; Eickhoff et al. 2005).

## Results

### Neural Responses to Evoked Versus Emitted Laughter

**Figure 1.**
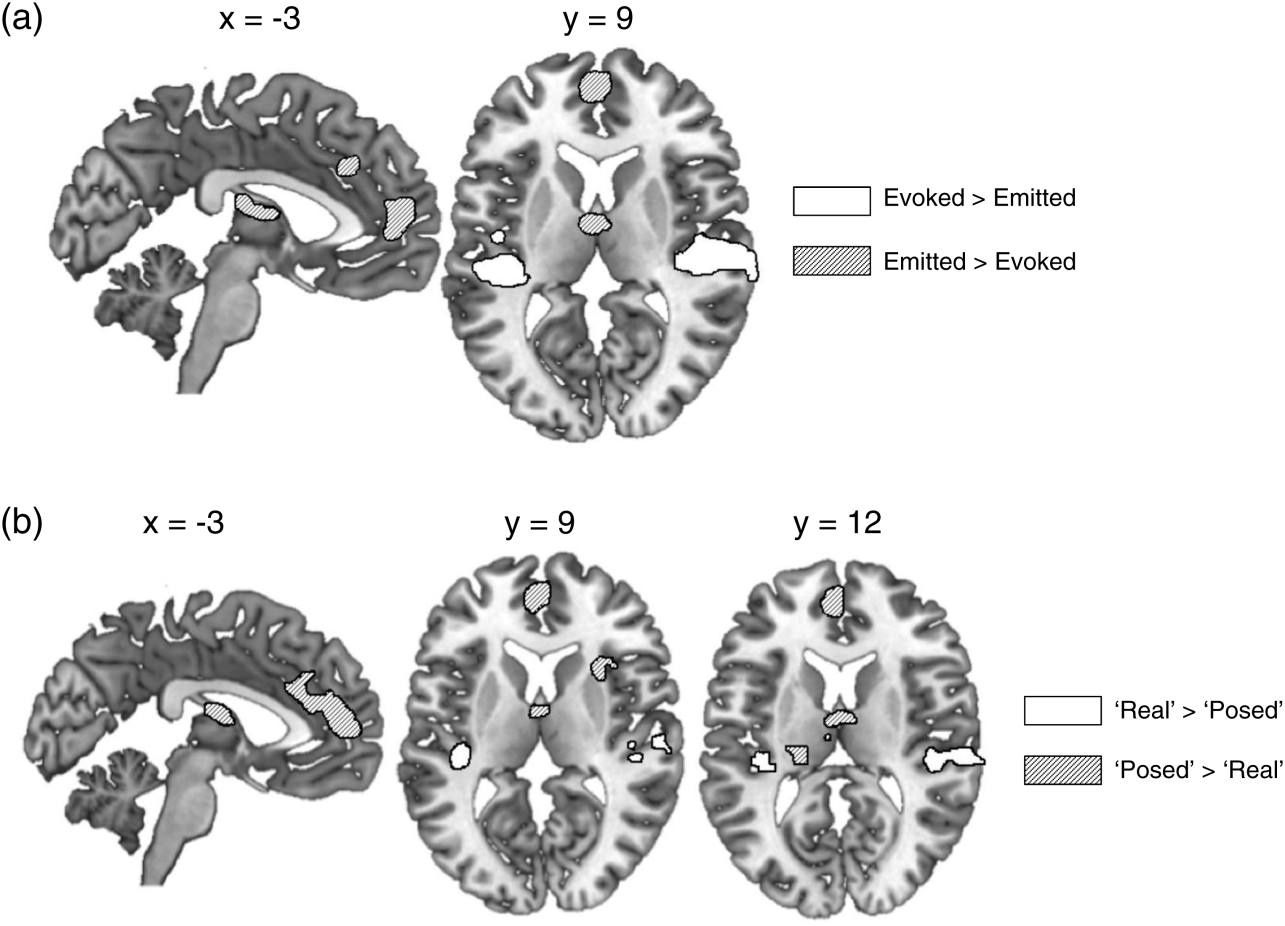
Direct comparison of Evoked and Emitted laughter, where (*a*) responses were coded according to their predefined categories or (*b*) according to each participant's post-test classification of the items as “Real” and “Posed.” Activations are shown at a voxel height threshold of *P* < 0.001 and a corrected cluster extent threshold of *P* < 0.001 (Slotnick et al. 2003).

The Evoked laughs gave greater activation than Emitted laughs in bilateral superior temporal gyrus (STG) and Heschl's gyrus (HG), while the converse contrast showed greater activation for the Emitted laughs in amPFC, anterior cingulate gyrus, and left thalamus (Fig. 1*a* and Table 1).

**Table 1:** Brain regions showing significantly different activation in response to Evoked/“Real” and Emitted/“Posed” laughter

Contrast	No. of voxels	Region	Coordinate			*T*	*Z*
			*x*	*y*	*z*		
Evoked > Emitted	258	Right superior temporal gyrus	48	−27	15	6.55	4.73
			60	−21	12	6.36	4.65
			48	−15	3	5.32	4.15
	153	Left superior temporal gyrus	−42	−27	9	6.93	4.89
			−54	−27	12	4.91	3.93
			−48	−9	3	4.19	3.51
Emitted > Evoked	51	Left superior medial gyrus	−3	54	9	5.52	4.26
	35	Left temporal thalamus	−3	−6	9	4.86	3.90
			6	−15	15	4.66	3.79
			−3	−15	12	4.38	3.62
	26	Right anterior cingulate cortex	0	30	30	4.38	3.62
			6	24	18	3.98	3.37
“Real” > “Posed”	44	Right superior temporal gyrus	45	−27	12	5.51	4.25
	33	Left Heschl's gyrus	60	−24	12	4.76	3.84
			−39	−27	9	5.56	4.27
			−42	−21	0	4.31	3.58
“Posed” > “Real”	152	Left superior medial gyrus, left/right anterior cingulate cortex	−3	54	9	5.51	4.25
			3	30	21	4.85	3.90
			12	36	27	4.79	3.86
	80	Right middle frontal gyrus	39	33	39	5.79	4.39
			33	30	48	4.35	3.61
			33	24	33	3.73	3.21
			36	21	6	4.39	3.63
	76	Left temporal thalamus	−21	−24	12	5.04	4.00
			−9	−15	18	4.38	3.62
			−3	−9	15	4.24	3.54
	30	Right putamen/insula	27	18	6	5.13	4.05

The contrasts Evoked/“Real” Laughter > Emitted/“Posed” Laughter and Emitted/“Posed” Laughter > Evoked/“Real” Laughter are reported at a voxel height threshold of *P* < 0.001 (uncorrected), and a corrected cluster threshold of *P* < 0.001 (Slotnick et al. 2003). Coordinates are in Montreal Neurological Institute (MNI) stereotactic space.

In order to more directly explore the contrast of perceived emotional authenticity, the first-level (single-subject) model was reanalyzed with the Evoked and Emitted conditions now recategorized as “Real” and “Posed,” respectively, according to the individual participants' classification responses in the behavioral post-test. These recoded group comparisons of “Real” and “Posed” laughs contrast revealed largely similar activations as obtained in the contrast of the predefined conditions Evoked and Emitted (Fig. 1*b* and Table 1). Despite some numerical differences in cluster sizes across the original and recoded analyses, a direct comparison of the Evoked versus Emitted and “Real” versus “Posed” contrasts identified no significant differences between the 2 models.

### Individual Differences in Detecting Emotional Authenticity

**Figure 2.**
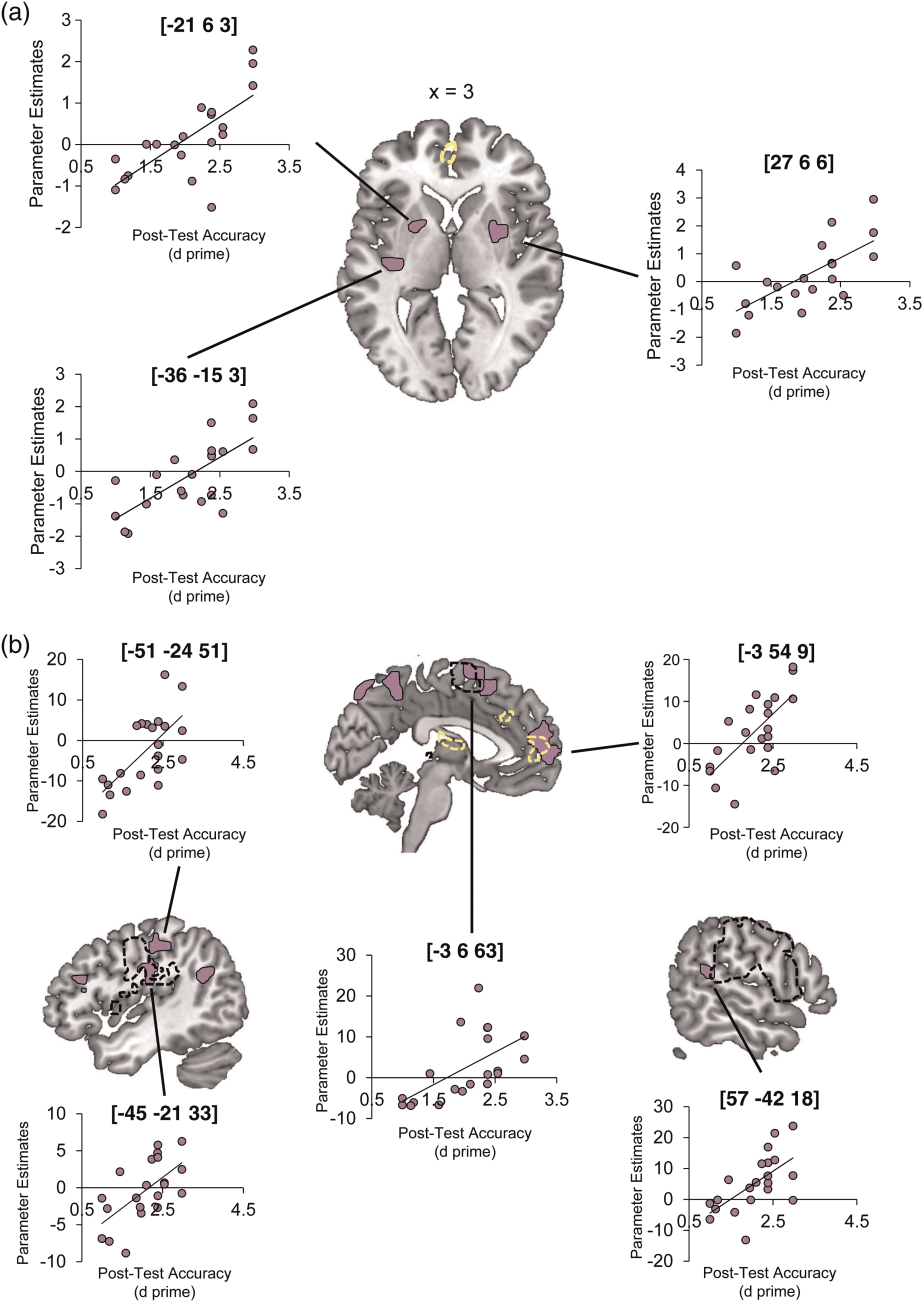
Relationship between neural responses to laughter and post-test classification of stimuli as “real” or “posed.” Images show significant clusters (purple shading) from regression analyses using individual post-test scores on the classification as a predictor of the BOLD response for the contrasts (*a*) Emitted > Evoked laughter and (*b*) All Laughs (Evoked and Emitted) > Rest. The scatter plots show the relationship between the neural and behavioral data taken from local peaks in significantly active clusters within each model. Regression activations are shown at a voxel height threshold of *P* < 0.005, and a corrected cluster extent threshold of *P* < 0.001 (Slotnick et al. 2003), alongside (in *a*) the regions activated during smiling (compared with Rest, in black dashed outline at *P* < 0.001, uncorrected, no cluster extent threshold), and (in *a* and *b*) the main group contrast of Emitted > Evoked laughs (in yellow dashed outline at a voxel height threshold of *P* < 0.001, and a corrected cluster extent threshold of *P* < 0.001).

In an individual differences approach, whole-brain second-level regression analyses explored the predictive relationship between accuracy on the post-test and neural responses to laughter in the passive listening phase of the fMRI experiment. The behavioral post-test showed that the participants were able to classify the laughs into “Real” and “Posed” categories with a high degree of accuracy (mean accuracy: 82.5%, mean *d*′: 2.06). However, while all participants scored above chance (50%), there was a wide range of performance across individuals (accuracy: 69–93%, *d*′: 1.00–2.98). A separate regression model was run for each of Evoked > Emitted, Emitted > Evoked, and All Laughs (Evoked and Emitted) >Rest, using individual *d*′ scores as the predictor variable in each case. These analyses tested 2 hypotheses about the neural correlates of individual variability in laughter perception—first, that the behavioral ability to discriminate “Real” from “Posed” laughter should be expressed in the size of the differential neural response to the 2 laughter conditions (i.e., in the contrasts of Evoked vs. Emitted laughs) and second, that variability in behavior might be linked to more general processing mechanisms in brain regions engaged by all laughter vocalizations (i.e., that it should relate to the degree of activation in response to both Evoked and Emitted laughter). The regression analysis on the contrast Emitted > Evoked identified several sites in amPFC whose activation was positively correlated with behavioral performance, as well as a number of sites in the dorsal striatum, though none of these sites directly overlapped with the regions identified in the mean contrast of Emitted > Evoked (see Fig. 2*a* and Table 2). However, the regression on the contrast All Laughs > Rest revealed a larger cluster in amPFC that positively correlated with *d*′ and overlapped with the site identified in the main group contrast Emitted > Evoked. With the proviso that there may have been greater overall variability in the All Laughs > Rest contrast with which to detect significant effects, this suggests that the passive engagement of mentalizing processes in amPFC occurs in response to all laughter vocalizations, and that the extent to which these processes are engaged—despite no overt task demands—is positively related to successful judgments of emotional stimuli. In addition to the amPFC, clusters positively related to behavioral performance were identified in left pre-SMA, left somatosensory cortex, and right supramarginal gyrus, all of which overlapped with the regions activated in the orofacial movements localizer contrast of Smiling > Rest (see Fig. 2). Table 3 lists all the significant clusters identified in the regression analyses. There were no significant positive activations in the regression model examining individual differences in the contrast of Evoked > Emitted laughs.

**Table 2:** Neural responses related to successful detection of emotional authenticity

Contrast	No. of voxels	Region	Coordinate			*T*	*Z*
			*x*	*y*	*z*		
All Laughs > Rest	*205*	*Left/right superior medial gyrus/anterior cingulate cortex*	*−6*	*54*	*9*	*5.16*	*4.03*
			*−6*	*51*	*18*	*3.97*	*3.35*
			*6*	*57*	*15*	*3.85*	*3.27*
	154	Left/right pre−/cuneus	*−*9	*−*78	54	4.58	3.71
			*−*3	*−*78	48	3.80	3.24
			−3	−54	57	3.67	3.15
	121	*Left pre-SMA/superior frontal gyrus (Brodmann Area 6)*	*−12*	*24*	*57*	*3.98*	*3.35*
			*−3*	*18*	*51*	*3.77*	*3.22*
			*−3*	*6*	*63*	*3.66*	*3.14*
	66	Left postcentral gyrus (Brodmann Areas 2, 1, 3, 4)	−51	−24	51	3.99	3.36
	57	Left middle frontal gyrus	−21	48	27	3.68	3.16
	50	Left angular gyrus	−39	−60	27	4.10	3.43
	46	Left superior temporal sulcus	−66	−42	6	5.25	4.08
	41	Right superior temporal sulcus	45	−30	−6	5.81	4.35
			54	−9	−21	4.17	3.47
			48	−21	−9	3.55	3.07
	41	Left insula	−30	21	12	4.14	3.45
	34	Left middle frontal gyrus	−30	3	57	4.02	3.38
	30	Left supramarginal gyrus	−63	−45	24	3.95	3.33
	*24*	*Left postcentral gyrus/Rolandic operculum (Brodmann Areas 3, 4)*	*−45*	*−21*	*33*	*3.79*	*3.23*
			*−45*	*−21*	*21*	*3.24*	*2.86*
			*−45*	*−12*	*30*	*3.22*	*2.84*
	23	Left inferior frontal gyrus (pars triang.; Brodmann Area 45)	−48	30	18	3.66	3.14
			−48	27	24	2.95	2.64
	20	*Right STG/supramarginal gyrus*	57	−42	24	3.41	2.98
			51	−45	15	3.34	2.93
Emitted > Evoked	57	Right superior medial gyrus	9	51	39	4.29	3.54
			12	39	42	3.90	3.30
			6	57	33	3.87	3.28
	41	Left middle/superior frontal gyrus	−24	21	39	4.57	3.71
			−15	6	48	4.25	3.52
			−24	12	45	3.53	3.06
	35	Right putamen	27	6	6	4.01	3.37
	32	Left insula/Heschl's gyrus	−36	−15	3	4.33	3.57
			−39	−24	12	3.20	2.83
	30	Right anterior cingulate cortex	12	39	24	4.50	3.67
	25	Left putamen	−21	6	3	4.35	3.58
			−18	3	−12	3.66	3.15
			−21	15	0	3.05	2.72
	23	Left superior medial/frontal gyrus	−6	51	45	3.64	3.13
			−15	42	42	3.29	2.89
			−3	48	36	3.09	2.75
	21	Left superior frontal gyrus	−15	27	51	3.83	3.25
			−15	27	42	3.57	3.09

The table lists the results of regression analyses of behavioral classification accuracy against the responses to the contrast Emitted laughter > Evoked laughter, and the contrast All Laughs > Rest. Significant clusters in prefrontal and sensorimotor cortex taken forward into connectivity analyses are italicized. Results are reported at a voxel height threshold of *P* < 0.005 (uncorrected), and a corrected cluster threshold of *P* < 0.001 (Slotnick et al. 2003). Coordinates are in Montreal Neurological Institute (MNI) stereotactic space.

SMA, supplementary motor area; pars triang., pars triangularis; STG, superior temporal gyrus.

**Table 3:** Brain regions showing significant positive psychophysiological interactions (PPIs) with sensorimotor responses to laughter, dependent on the contrast of “Real” > “Posed”

Seed region	No. of voxels	Target region	Coordinate			*T*	*Z*
			*x*	*y*	*z*		
Left pre-SMA	96	Left/right pre-SMA (Brodmann Area 6)	0	6	48	4.36	3.61
	48	Left cuneus	−15	−57	24	4.98	3.97
	46	Left caudate nucleus	−18	−6	24	4.02	3.40
			−15	0	18	3.85	3.29
	42	Right precuneus	6	−54	48	4.01	3.39
	32	Left/right paracentral lobule (Primary motor cortex and SMA; Brodmann Areas 4, 6)	−3	−30	60	3.11	2.77
			6	−24	66	3.58	3.11
	28	Left postcentral gyrus (Brodmann Areas 3, 4, 6)	−42	−21	51	3.95	3.36
	25	Left cerebellum (Lobule V)	−6	−57	−9	4.48	3.68
			0	−57	−3	4.17	3.50
Left postcentral gyrus	95	Right middle/inferior frontal gyrus	42	39	30	4.73	3.83
			48	33	27	4.41	3.64
			39	36	21	3.93	3.34
	41	Right superior occipital cortex/cuneus	24	−78	45	3.44	3.01
			18	−78	33	3.34	2.94
			27	−75	30	2.90	2.62
	36	Left precentral gyrus (Brodmann Area 6)	−39	3	42	3.79	3.25
			−42	−6	39	3.52	3.07
	28	Left parietal operculum	−57	−30	15	3.80	3.26
Right supramarginal gyrus	108	Left/right paracentral lobule (Primary motor cortex and SMA; Brodmann Areas 4, 6)	0	−27	60	4.22	3.53
			0	−18	69	3.74	3.21
			−6	−33	51	3.20	2.84
	25	Left inferior parietal lobule	−57	−39	48	4.03	3.41
			−45	−45	35	3.93	3.35
			−51	−45	42	3.49	3.05
	23	Left precentral/superior frontal gyrus (Brodmann Area 6)	−18	−18	69	3.94	3.35
			−24	−3	69	3.35	2.95

Reported at a voxel height threshold of *P* < 0.005 (uncorrected), and a corrected cluster threshold of *P* < 0.001 (Slotnick et al. 2003). Coordinates are in Montreal Neurological Institute (MNI) stereotactic space.

SMA, supplementary motor area.

### Modulation of Functional Connections by Perceived Emotional Authenticity

**Figure 3.**
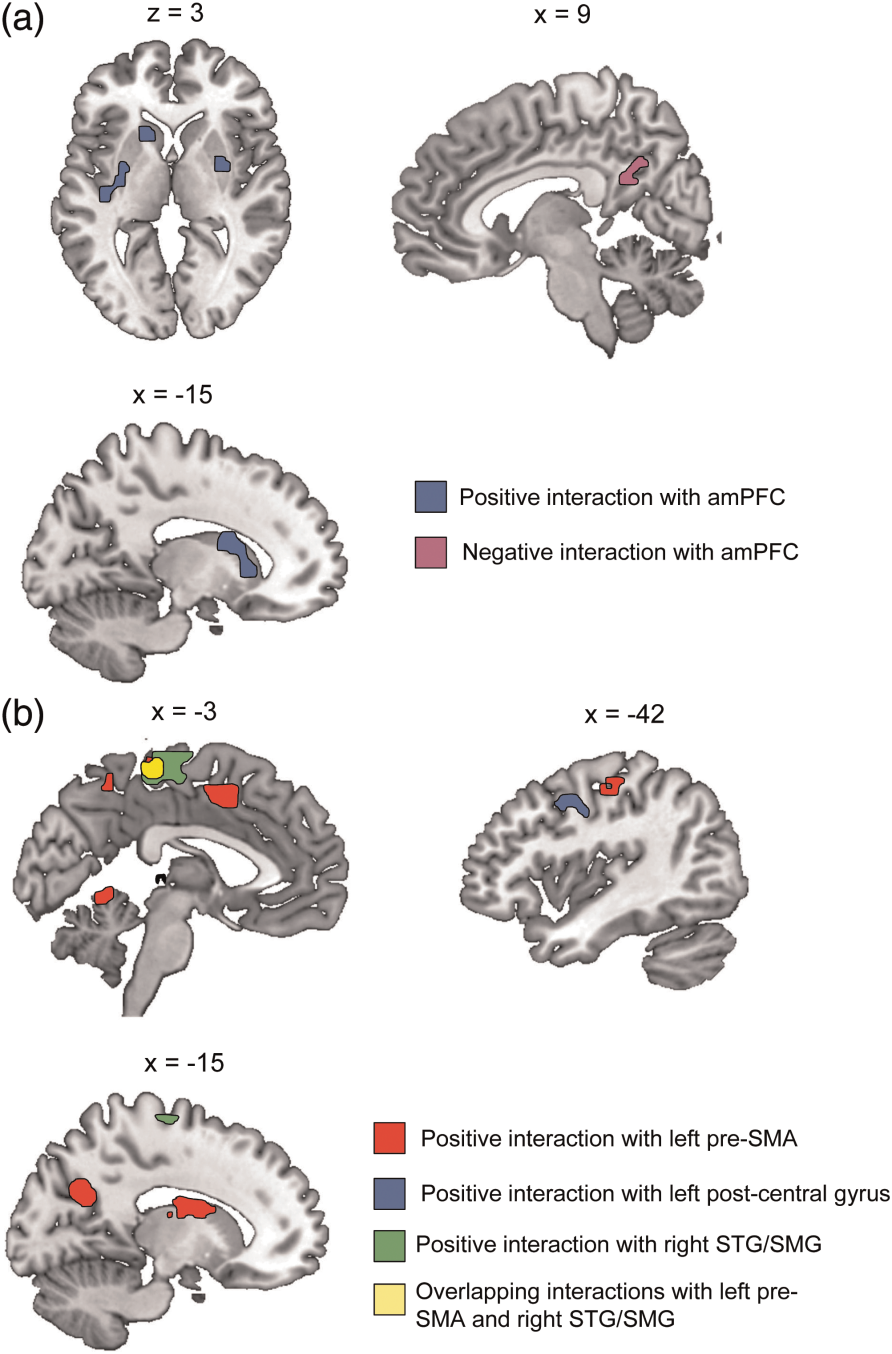
Differing functional connectivity dependent on perceived emotional authenticity of heard laughter. (*a*) Images show regions that exhibited positive interactions during the perception of “Real” laughter (compared with “Posed”) with the medial prefrontal activation identified in the individual differences regression on All Laughs > Rest (see Fig. 2*b*). amPFC = anterior medial prefrontal cortex. (*b*) Images show regions that exhibited modulations in connectivity during the perception of “Real” laughter (compared with “Posed”) with the sensorimotor regions identified in the individual differences regression analysis on All Laughs > Rest (see Fig. 2*b*). Activations are shown at a voxel height threshold of *P* < 0.005 and a corrected cluster extent threshold of *P* < 0.001 (Slotnick et al. 2003). STG, superior temporal gyrus; SMG, supramarginal gyrus.

Based on our hypothesis regarding a role for sensorimotor cortex in laughter perception, a functional connectivity analysis explored the interactions between 3 sensorimotor regions and activity in the rest of the brain that might be modulated by the perceived authenticity of laughter. This was particularly motivated by the observation that these sensorimotor sites were associated with variability in behavioral performance, yet did not show the hypothesized enhanced response to the Evoked/“Real” laughter compared with the Emitted/“Posed” laughter tokens (even at reduced thresholds). To this end, group PPI analyses were run to explore changes in connectivity across the “Real” and “Posed” laughter conditions (recoded using the individual post-test responses), using as seed regions the clusters in left postcentral gyrus, left pre-SMA, and right posterior SMG identified in the regression of *d*′ on All Laughs > Rest (and which overlapped with the regions activated by the orofacial movements localizer). An additional analysis explored whole-brain interactions with the amPFC cluster identified in the individual differences regression on All Laughs > Rest (and which was also implicated in mean differences between Emitted/“Posed” and Evoked/“Real” laughter—see Fig. 1). The PPI analyses revealed a set of significant positive interactions from all 4 seed regions—that is, target regions were identified that showed more strongly positive correlations with the seed regions during “Real” laughs compared with “Posed” laughter. For the sensorimotor seeds, several significant interacting target sites were located in other regions of sensorimotor cortex, including left precentral gyrus, left postcentral gyrus, SMA/medial primary motor cortex, as well as cerebellum and sites in the dorsal striatum (see Fig. 3*a* and Table 3). The amPFC seed region also showed positive interactions dependent on the contrast “Real” > “Posed” with striatal target sites in the caudate, insula, and putamen, and a negative interaction (i.e., stronger connectivity for “Posed” > “Real”) with right precuneus (see Fig. 3*b* and Table 4).

**Table 4:** Brain regions showing significant positive psychophysiological interactions (PPIs) with medial prefrontal responses to laughter, dependent on the contrasts of “Real” > “Posed” and “Posed” > “Real”

Contrast	No. of voxels	Target region	Coordinate			*T*	*Z*
			*x*	*y*	*z*		
“Real” > “Posed”	60	Left insula/putamen	−27	−6	6	6.88	4.87
			−39	−15	3	4.28	3.57
	56	Left caudate nucleus	−15	6	15	5.40	4.19
			−15	15	12	4.62	3.77
			−15	18	3	4.59	3.75
	26	Right putamen	30	−3	6	4.66	3.79
“Posed” > “Real”	48	Right/left precuneus	6	−51	27	3.82	3.27
			9	−57	36	3.64	3.15
			0	−63	27	3.17	2.82

Reported at a voxel height threshold of *P* < 0.005 (uncorrected), and a corrected cluster threshold of *P* < 0.001 (Slotnick et al. 2003). Coordinates are in Montreal Neurological Institute (MNI) stereotactic space.

## Discussion

The current study set out with 2 main aims. The first was to identify regions responding to the passive perception of emotional authenticity in heard laughter. Here, we identified a set of cortical and subcortical regions that automatically distinguished between authentic and acted laughs, and showed that this pattern held whether the laughter conditions were coded according to the context in which they were produced—Evoked vs. Emitted—or the participants' post hoc evaluations of the laughs as “Real” or “Posed.” Our second aim was to explore whether sensorimotor responses to heard laughter would be modulated by contagiousness, through the comparison of Evoked and Emitted laughter, which significantly differed on measures of motoric and emotional infectiousness. Despite finding no significant enhancement in sensorimotor responses to the more contagious laughter, an individual differences analysis revealed that activation of pre-SMA and lateral somatosensory cortex to all laughter, regardless of authenticity, was positively correlated across individuals with accuracy in classification of Evoked and Emitted laughs in a post-test. These sensorimotor sites showed functional connections with several cortical and subcortical sites that were modulated by the perceived authenticity of laughter vocalizations. Thus, we have shown a role for sensorimotor cortex not limited to a basic behavioral reflex, as predicted, but as part of a whole-brain mechanism for the successful evaluation and understanding of emotional vocalizations. We discuss the findings in detail below.

### Passive Responses to Emotional Authenticity in Heard Laughter

During passive listening, amPFC and anterior cingulate cortex were engaged more strongly for Emitted than Evoked laughter. This indicates stronger engagement of mentalizing processes in response to the Emitted laughter (Frith and Frith 2006, 2010; Lewis et al. 2011), presumably reflecting an obligatory attempt to determine the emotional state and intentions of the laugher. Kober et al. (2008) identify several possible roles for medial prefrontal sites in emotion perception, including the attribution of mental states in oneself or others, and in metacognitive processing of affective inputs (e.g., to generate or regulate emotion; Mitchell and Greening 2012; Phillips et al. 2003). The current data do not allow us to easily tease these 2 apart. We note that it is unlikely that emotion regulation would be more strongly engaged for the Emitted items, as these were rated lower overall on scales of Arousal, Intensity, and Emotional and Behavioral Contagion.

A comparison of “Real” with “Posed” laughter, where the laughter categories were redefined in each participant according to how they labeled the laughs in the behavioral post-test, identified similar patterns of activation implicating amPFC, anterior cingulate cortex, thalamus, and dorsal striatum in a preferential response to laughter perceived as nongenuine. Finally, the regression analyses found that individual accuracy scores on a post-test categorization of Evoked and Emitted laughs as “Real” and “Posed” positively predicted the degree of activation of amPFC (as well as precuneus, which has also been implicated in a mentalizing network; Van Overwalle and Baetens 2009) during passive listening. This consistency in results relating mentalizing regions of cortex to passively heard posed laughter provides additional support for good alignment between how the Evoked and Emitted conditions were designed and produced with how they were perceived by the fMRI participants.

A previous study identified greater activation of medial prefrontal cortex (including anterior cingulate cortex) and precuneus during listening to “emotional” laughter (e.g., taunting, joyful) compared with laughter produced by tickling, and greater activation of STG for the tickling laughs in the converse comparison (Szameitat et al. 2010). We identify a similar profile of activations, but suggest that it is the social-emotional ambiguity of the Emitted laughter that leads to the stronger engagement of mentalizing processes, rather than the complexity of the speaker's emotional state. Although reaction times were not recorded in the current experiment, these could indicate whether the Emitted laughter might have engaged additional decision-making processes to resolve this emotional ambiguity (as demonstrated in a recent EEG experiment; Calvo et al. 2013). Our Evoked laughs were not reflexive responses to touch, but rather elicited through the complex process of humor appreciation leading to a positive emotional state. As Provine (1996; 2000) points out, the experience of humor in humans has a strong social basis—we tend not to laugh when alone, but when we do, it tends to be while viewing or listening to other humans (e.g., in a movie) or thinking about events involving other people. By the same token, we do not suggest that the Emitted tokens were unemotional. Davila-Ross et al. (2011) showed that the onset latencies of laughter-elicited laughter in chimpanzees fell into 2 populations, 1 rapid (more characteristic of automatic, affective vocalization) and 1 delayed, and the authors suggest that this may reflect a mixture of nonautomatic and affective processes underlying the laughter behavior. The Emitted samples in the current experiment may also reflect such combinations, leading to increased ambiguity for the perceiver.

A recent fMRI study compared the perceptual responses to authentic and “play-acted” emotional speech expressing a range of positive and negative emotions, and identified sensitivity to authenticity in medial prefrontal cortex (Drolet et al. 2012). However, they found *increased* activation in medial prefrontal cortex for authentic stimuli compared with acted tokens. The authors suggest that their authentic stimuli, which were first-person accounts of emotional life events taken from radio interview archives, were more likely to activate the listener's own autobiographical memories of emotional experiences than the acted tokens. In this sense, they claim their authentic recordings were socially more “open-ended” and thus engaged mentalizing processes as the listener attempted to establish the speaker's intentions in light of their own past experience. This is in line with our interpretation of greater ambiguity in the Emitted laughs compared with the Evoked tokens in the current experiment.

### The Role of Sensorimotor Cortex in Laughter Perception

Hearing laughter frequently elicits laughing from the listener (Provine 1992), and positive emotional vocalizations such as laughter and cheers of triumph have previously been shown to preferentially engage parts of the cortical sensorimotor system used for smiling and laughing (Warren et al. 2006), supporting the view that there is a basic sound-to-action response to emotional vocalizations that tend to be performed in groups. Humans are primed to echo the laughter we hear, whether or not we are sharing the emotional experience of the laughers with whom we are joining in. In the current study, we predicted that motor and somatosensory cortical fields would be more strongly engaged by more contagious laughter, that is, by the Evoked stimuli. Although sensorimotor regions did not show a greater mean response to the Evoked/Real compared with the Emitted/Posed laughs, regions in pre-SMA and lateral somatosensory cortex showed a graded response to all laughter that could be predicted by the participant's postscan accuracy on emotional authenticity judgments. Thus, we find that a sensorimotor response to positive emotional vocalizations, in this case laughter, does not reflect a simple readiness to join in, but rather acts as part of a mechanism for the emotional interpretation and understanding of these sounds. Further, these responses occurred automatically, in the absence of a task or explicit instruction about the presence of different types of laughter in the experiment (cf., Drolet et al. 2012; Szameitat et al. 2010, in which the listeners were informed in advance of the experimental manipulations and performed active tasks in the scanner). Our current finding suggests that there is a behavioral benefit associated with recruiting sensorimotor cortex when listening to laughter (in this case, enhanced accuracy in evaluating the emotional authenticity of laughs), rather than a basic motor priming associated with the tendency to “echo” heard laughs. Studies using transcranial magnetic stimulation (TMS) to disrupt processing in somatosensory cortex have shown that this impairs performance on the discrimination of emotional faces (Pitcher et al. 2008) and vocalizations (Banissy et al. 2010). A number of studies have further linked variability in cortical motor and somatosensory activations to individual differences in socially relevant personality traits, such as empathy, both for the perception of action (Gazzola et al. 2006) and for mirror touch (Banissy and Ward 2007). Our finding offers a candidate functional role for this link: the obligatory, automatic recruitment of sensorimotor cortex when listening to laughter is associated with better performance at distinguishing authentic mirthful laughter from deliberate, acted laughs. The link between empathy and the greater engagement of sensorimotor systems may reflect an enhanced simulation mechanism for social understanding (Adolphs et al. 2000; Adolphs 2002, 2009; Carr et al. 2003; Hooker et al. 2008)—in support of this idea, Germine et al. (2011) demonstrated that participants with high social anhedonia showed a lower enhancement of responses in somatosensory cortex (and amPFC) during an emotional face discrimination task, compared with control visual discriminations.

### Interacting Systems in the Perception of Laughter–Sensorimotor, Cognitive, and Emotional Networks

We observed differential network engagement for the 2 laughter categories, where the laughs perceived as “real” were accompanied by a more positive correlation between sensorimotor sites and a range of cortical and subcortical regions. Several of the significant clusters in the individual differences and PPI analyses parallel the correlates of voluntary (posed) and involuntary (ticklish) laughter production reported in recent work by Wattendorf et al. (2012), which included SMA, Rolandic and parietal operculi, the putamen, insula, and cerebellum. Wattendorf et al. showed that activation in extensive parts of the laughter production network was similar whether the task was to produce voluntary or involuntary laughs, or to suppress the desire to laugh during tickling. Our finding of modulations in connectivity between sensorimotor sites and other brain regions dependent on the laughter condition was particularly illuminating, given that we did not observe the predicted enhancement in the mean sensorimotor cortical responses to the perception of Evoked/Real laughs compared with Emitted/Posed laughs. The results of this connectivity analysis offered additional support for a mechanistic role for sensorimotor regions in the social-emotional evaluation of heard vocalizations.

Wild et al. (2003) proposed that laughter is controlled by a network of cortical and subcortical sites, where the trigger to laugh comes from the periaqueductal gray and pontine reticular formation (which in turn receive inputs from cortex), and subcortical structures including the basal ganglia and hypothalamus. They identify motor and premotor cortex as inhibitory nodes acting, via the cerebellum, on the mesencephalic “laughter centers,” and proposed that laughter occurs when these cortical sites release their inhibition to allow vocalization. In the context of the current experiment, greater voluntary inhibition of a laughter response may be required for the (more contagious) Evoked tokens, possibly implicating motor regions such as the pre-SMA and their interactions with subcortical structures in the dorsal striatum, all of which were repeatedly implicated in authenticity-relevant processing throughout the current dataset.

Interestingly, a connectivity analysis from a seed region in amPFC, which had already been identified as showing greater activation in the contrast “Posed” > “Real,” showed condition-dependent interactions with similar sites as identified using the sensorimotor seeds, including the dorsal striatum and precuneus. A recent study identified several emotion processing networks from a meta-analysis of 162 neuroimaging studies of emotion perception (Kober et al. 2008). The authors present an interesting view of the connectivity profiles of a “Medial Prefrontal Cortex group” (comprising dorsomedial prefrontal cortex and parts of anterior cingulate cortex), in comparison with those exhibited by a “Cognitive/Motor group” (including the inferior frontal gyri, right frontal operculum and left pre-SMA). Kober and coworkers report that both groups show connections with a “Lateral paralimbic group” (comprising insula, ventral striatum, posterior orbital gyrus, and temporal pole), but are not directly connected. Kober and coworkers propose that the medial prefrontal regions appear to be more strongly associated with affective processes of the limbic system rather than cognitive function, and that regions of dorsal and posterior insula offer a bridge between this system and the Cognitive/Motor group—the authors go on to suggest that the Medial Prefrontal Cortex group “interfaces between cognitive context and core affect” (p. 1016), while the Cognitive/Motor group may be concerned with cognitive control and the “context-appropriate selection of actions and attention for action” (p. 1014). The current dataset affords glimpses of these interacting networks in emotion perception, via the use of individual differences and connectivity analyses. A challenge for future work will be to establish greater details of the mechanism by which the cognitive and affective evaluations of heard laughter might take place—our findings (and those of Germine et al. 2011) indicate prominent roles for both medial prefrontal and sensorimotor systems.

A further challenge will be to determine how our findings can be related to the neural correlates of humor appreciation. Mobbs et al. (Mobbs et al. 2003) ran a study of humor perception, where the intensity of humorful experience was significantly correlated with activation in regions including pre-SMA, SMA, anterior cingulate, and putamen, some of which we also see in functional interactions with the sensorimotor seed regions in the current experiment. Mobbs et al. relate SMA to the outward production of laughter, but also suggest that it could, in conjunction with the dorsal anterior cingulate cortex, be involved in reward-based decision-making via dopaminergic connections with the ventral striatum (see also Bekinschtein et al. 2011). Stimulation of midline cortical regions (including anterior SMA) has been associated with the behavioral initiation of laughter and vocalizations in humans and other animals (Fried et al. 1998; Jurgens 2002; Burgdorf et al. 2007). Thus, the recurring involvement of SMA and ACC, as well as striatal structures, in the current study may reflect both sensorimotor and emotional aspects of the contagiousness of heard laughter.

Fredrickson (1998) suggested that “psychologists have inadvertently marginalized the emotions … that share a pleasant subjective feel” (p. 300), and here we have demonstrated the advantage of using a strongly positive emotion to probe neural networks evaluating emotional authenticity in vocalizations. We demonstrate that variation in cortical motor/somatosensory systems reflects the efficiency of a whole-brain system that processes the social and emotional relevance of heard laughter and regulates the listener's behavioral response to the stimulus.

## Conclusions

This is the first study to directly compare the neural correlates of involuntary/authentic and voluntary nonverbal expressions of emotion in the voice. We report that Evoked and Emitted laughter show distinct cortical signatures in perception, consistent with their different roles in social interactions in humans. We have extended our previous finding of a sensorimotor role in laughter perception to show that greater activation of cortical motor and somatosensory regions is related to greater acuity in distinguishing “real” and “posed” laughs. Our results therefore demonstrate robust and obligatory processing of authenticity in heard laughter, and suggest that sensorimotor links in emotional processing may support aspects of social understanding.

## Funding

This work was supported by a Wellcome Trust Senior Research Fellowship (WT090961MA) awarded to S.K.S. Funding to pay the Open Access publication charges for this article was provided by The Wellcome Trust.

## References

[BHT227C1] Adolphs R (2002). Neural systems for recognizing emotion. Curr Opin Neurobiol.

[BHT227C2] Adolphs R (2009). The social brain: neural basis of social knowledge. Annu Rev Psychol.

[BHT227C3] Adolphs R, Damasio H, Tranel D, Cooper G, Damasio AR (2000). A role for somatosensory cortices in the visual recognition of emotion as revealed by three-dimensional lesion mapping. J Neurosci.

[BHT227C4] Banissy MJ, Sauter DA, Ward J, Warren JE, Walsh V, Scott SK (2010). Suppressing sensorimotor activity modulates the discrimination of auditory emotions but not speaker identity. J Neurosci.

[BHT227C5] Banissy MJ, Ward J (2007). Mirror-touch synesthesia is linked with empathy. Nat Neurosci.

[BHT227C6] Bekinschtein TA, Davis MH, Rodd JM, Owen AM (2011). Why clowns taste funny: the relationship between humor and semantic ambiguity. J Neurosci.

[BHT227C7] Blesser B (1972). Speech perception under conditions of spectral transformation. I. Phonetic characteristics. J Speech Hear Res.

[BHT227C8] Boersma P, Weenink D http://www.praat.org/.

[BHT227C56] Brainard DH (1997). The Psychophysics Toolbox. Spatial Vision.

[BHT227C9] Brett M, Anton JL, Valabregue R, Poline JB (2002). Region of interest analysis using an SPM toolbox. International Conference on Functional Mapping of the Human Brain.

[BHT227C10] Brueck C, Kreifelts B, Wildgruber D (2011). Emotional voices in context: a neurobiological model of multimodal affective information processing. Phys Life Rev.

[BHT227C11] Burgdorf J, Wood PL, Kroes RA, Moskal JR, Panksepp J (2007). Neurobiology of 50-kHz ultrasonic vocalizations in rats: electrode mapping, lesion, and pharmacology studies. Behav Brain Res.

[BHT227C12] Calvo MG, Marrero H, Beltrain D (2013). When does the brain distinguish between genuine and ambiguous smiles? An ERP study. Brain Cogn.

[BHT227C13] Carr L, Iacoboni M, Dubeau MC, Mazziotta JC, Lenzi GL (2003). Neural mechanisms of empathy in humans: a relay from neural systems for imitation to limbic areas. Proc Natl Acad Sci U S A.

[BHT227C14] Davila-Ross M, Allcock B, Thomas C, Bard KA (2011). Aping expressions? Chimpanzees produce distinct laugh types when responding to laughter of others. Emotion.

[BHT227C15] Drolet M, Schubotz RI, Fischer J (2012). Authenticity affects the recognition of emotions in speech: behavioral and fMRI evidence. Cogn Affect Behav Neurosci.

[BHT227C16] Edmister WB, Talavage TM, Ledden PJ, Weisskoff RM (1999). Improved auditory cortex imaging using clustered volume acquisitions. Hum Brain Mapp.

[BHT227C17] Eickhoff SB, Stephan KE, Mohlberg H, Grefkes C, Fink GR, Amunts K, Zilles K (2005). A new SPM toolbox for combining probabilistic cytoarchitectonic maps and functional imaging data. Neuroimage.

[BHT227C18] Fredrickson BL (1998). What good are positive emotions?. Rev Gen Psychol.

[BHT227C19] Fried I, Wilson CL, MacDonald KA, Behnke EJ (1998). Electric current stimulates laughter. Nature.

[BHT227C20] Frith CD, Frith U (2006). The neural basis of mentalizing. Neuron.

[BHT227C21] Frith U, Frith C (2010). The social brain: allowing humans to boldly go where no other species has been. Philos Trans Royal Soc B Biol Sci.

[BHT227C22] Gazzola V, Aziz-Zadeh L, Keysers C (2006). Empathy and the somatotopic auditory mirror system in humans. Curr Biol.

[BHT227C23] Germine LT, Garrido L, Bruce L, Hooker C (2011). Social anhedonia is associated with neural abnormalities during face emotion processing. Neuroimage.

[BHT227C24] Gervais M, Wilson DS (2005). The evolution and functions of laughter and humor: A synthetic approach. Q Rev Biol.

[BHT227C25] Hall DA, Haggard MP, Akeroyd MA, Palmer AR, Summerfield AQ, Elliott MR, Gurney EM, Bowtell RW (1999). “Sparse” temporal sampling in auditory fMRI. Hum Brain Mapp.

[BHT227C26] Hooker CI, Verosky SC, Germine LT, Knight RT, D'Esposito M (2008). Mentalizing about emotion and its relationship to empathy. Soc Cogn Affect Neurosci.

[BHT227C27] Jurgens U (2002). Neural pathways underlying vocal control. Neurosci Biobehav Rev.

[BHT227C28] Kober H, Barrett LF, Joseph J, Bliss-Moreau E, Lindquist K, Wager TD (2008). Functional grouping and cortical-subcortical interactions in emotion: a meta-analysis of neuroimaging studies. Neuroimage.

[BHT227C29] Lewis PA, Rezaie R, Brown R, Roberts N, Dunbar RIM (2011). Ventromedial prefrontal volume predicts understanding of others and social network size. Neuroimage.

[BHT227C30] Mitchell DG, Greening SG (2012). Conscious perception of emotional stimuli: brain mechanisms. Neuroscientist.

[BHT227C31] Mobbs D, Greicius MD, Abdel-Azim E, Menon V, Reiss AL (2003). Humor modulates the mesolimbic reward centers. Neuron.

[BHT227C32] Panksepp A (2000). The riddle of laughter: neural and psychoevolutionary underpinnings of joy. Curr Dir Psychol Sci.

[BHT227C33] Panksepp J (2005). Beyond a joke: from animal laughter to human joy?. Science.

[BHT227C34] Panksepp J, Burgdorf J (2003). “Laughing” rats and the evolutionary antecedents of human joy?. Physiol Behav.

[BHT227C35] Panksepp J, Burgdorf J, Hameroff SRKAWCDJ (2000). Laughing rats? Playful tickling arouses high-frequency ultrasonic chirping in young rodents. Toward a science of consciousness III: the third Tucson discussions and debates.

[BHT227C36] Phillips ML, Drevets WC, Rauch SL, Lane R (2003). Neurobiology of emotion perception I: the neural basis of normal emotion perception. Biol Psychiatry.

[BHT227C37] Pitcher D, Garrido L, Walsh V, Duchaine BC (2008). Transcranial magnetic stimulation disrupts the perception and embodiment of facial expressions. J Neurosci.

[BHT227C38] Provine RR (1992). Contagious laughter—laughter is a sufficient stimulus for laughs and smiles. Bull Psychon Soc.

[BHT227C39] Provine RR (1996). Laughter. Am Sci.

[BHT227C40] Provine RR (2000). Laughter: a scientific investigation.

[BHT227C41] Ross MD, Owren MJ, Zimmermann E (2010). The evolution of laughter in great apes and humans. Commun Integr Biol.

[BHT227C42] Ross MD, Owren MJ, Zimmermann E (2009). Reconstructing the evolution of laughter in great apes and humans. Curr Biol.

[BHT227C43] Sauter DA, Eisner F, Ekman P, Scott SK (2010). Cross-cultural recognition of basic emotions through nonverbal emotional vocalizations. Proc Natl Acad Sci USA.

[BHT227C44] Scott SK (2013). Laughter—the ordinary and the extraordinary. Psychologist.

[BHT227C45] Slotnick SD, Moo LR, Segal JB, Hart J (2003). Distinct prefrontal cortex activity associated with item memory and source memory for visual shapes. Cogn Brain Res.

[BHT227C46] Smoski MJ, Bachorowski JA (2003). Antiphonal laughter in developing friendships. Ann N Y Acad Sci.

[BHT227C47] Szameitat DP, Alter K, Szameitat AJ, Darwin CJ, Wildgruber D, Dietrich S, Sterr A (2009a). Differentiation of emotions in laughter at the behavioral level. Emotion.

[BHT227C48] Szameitat DP, Alter K, Szameitat AJ, Wildgruber D, Sterr A, Darwin CJ (2009b). Acoustic profiles of distinct emotional expressions in laughter. J Acoust Soc Am.

[BHT227C49] Szameitat DP, Darwin CJ, Wildgruber D, Alter K, Szameitat AJ (2011). Acoustic correlates of emotional dimensions in laughter: arousal, dominance, and valence. Cognition & Emotion.

[BHT227C50] Szameitat DP, Kreifelts B, Alter K, Szameitat AJ, Sterr A, Grodd W, Wildgruber D (2010). It is not always tickling: distinct cerebral responses during perception of different laughter types. Neuroimage.

[BHT227C51] Van Overwalle F, Baetens K (2009). Understanding others' actions and goals by mirror and mentalizing systems: a meta-analysis. Neuroimage.

[BHT227C52] Vettin J, Todt D (2004). Laughter in conversation: features of occurrence and acoustic structure. J Nonverbal Behav.

[BHT227C53] Warren JE, Sauter DA, Eisner F, Wiland J, Dresner MA, Wise RJS, Rosen S, Scott SK (2006). Positive emotions preferentially engage an auditory-motor “mirror” system. J Neurosci.

[BHT227C54] Wattendorf E, Westermann B, Fiedler K, Kaza E, Lotze M, Celio MR (2012). Exploration of the neural correlates of ticklish laughter by functional magnetic resonance imaging. Cereb Cortex.

[BHT227C55] Wild B, Rodden FA, Grodd W, Ruch W (2003). Neural correlates of laughter and humour. Brain.

